# DCR for nasolacrimal duct stenosis may be less effective than for complete obstruction

**DOI:** 10.1038/s41433-022-02048-9

**Published:** 2022-04-09

**Authors:** Yinon Shapira, Carmelo Macri, Eiman Usmani, Garry Davis, Dinesh Selva

**Affiliations:** 1 Discipline of Ophthalmology and Visual Science, University of Adelaide, Adelaide, SA Australia; 2 Department of Ophthalmology, Royal Adelaide Hospital and South Australian Institute of Ophthalmology, Adelaide, SA Australia

**Keywords:** Diseases, Quality of life

## Abstract

**Objectives:**

To ascertain the success of endo-DCR in nasolacrimal duct stenosis (NLDS) versus nasolacrimal duct obstruction (NLDO).

**Methods:**

Consecutive adult patients with epiphora attending a tertiary lacrimal clinic from February 2012 to February 2021 were reviewed. NLDS was diagnosed by patent lacrimal syringing and combined dacryocystography (NLD stenosis) and dacryoscintigraphy (post-sac delay) findings in all eyes. Cases with evidence of canalicular stenosis or other identifiable causes of epiphora were excluded. The epiphora resolution and improvement rates following endo-DCR were compared between NLDS and complete NLDO cases.

**Results:**

DCRs in 24 NLDS (23 patients, 69.6% females, mean age 61.0 ± 17.07) and 58 NLDO (56 patients, 69.6% females, mean age 61.9 ± 17.4) were included. Resolution of epiphora was achieved in 10 (41.7% [95% CI 0.24–0.61]) of the NLDS cases compared to 40 (69.0% [95% CI 0.56–0.79]) in NLDO (*p* = 0.021). Improvement of epiphora (i.e., either improvement or resolution) was noted in 17 (70.8% [95% CI 0.51–0.85]) of NLDS and 53 (91.4% [95% CI 0.81–0.96]) of NLDO cases (*p* = 0.034). Three patients (12.5%) with NLDS had subsequent lacrimal procedures (one DCR revision, two Jones tube) at a median of 14 (range 11–21) months. 71.4% of the NLDS patients responded to a phone questionnaire at a median of 93 months postoperatively. Of these, 46.7% reported resolution or significant improvement, and 33.3% reported slight improvement. 64.3% said they would recommend DCR to others suffering from epiphora.

**Conclusion:**

Endo-DCR may benefit approximately 70% of patients with NLDS. The success of endo-DCR in complete NLDO may be higher.

## Introduction

DCR, whether external or endoscopic, is well established as the standard procedure for treating complete nasolacrimal duct obstruction (NLDO), yielding high success rates [1–3]. However, patients with patent but dysfunctional NLD drainage are not uncommonly encountered in the lacrimal clinic [4, 5]. While these patients are often referred to as having ‘functional block’, using clinical assessment alone in their diagnosis (i.e., lacrimal syringing and/or Jones test) cannot reliably determine the potential existence of nasolacrimal duct stenosis (NLDS). The combination of dacryocystography (DCG) and dacryoscintigraphy (DSG) can clarify the specific type and degree of NLD drainage impairment [5, 6].

Previous studies that did not methodically distinguish between NLD stenosis and non-anatomical functional block report between 50–94% success rates of DCR in this combined group of patients [7–12]. We thus sought to audit our endoscopic DCR results in a homogenous cohort of anatomical NLDS, confirmed by combining DCG and DSG findings, so that we may better inform such patients of likely success in the future. Furthermore, we compared the outcomes in this group to those in patients with complete anatomical NLDO undergoing the same intervention.

## Methods

Data were collected retrospectively from consecutive adult patients with epiphora attending the Royal Adelaide Hospital lacrimal clinic from February 2012 to February 2021. The study received Institutional Review Board (IRB) approval and adhered to the tenets of the Declaration of Helsinki.

Patients with puncto-canalicular obstruction/stenosis, eyelid malposition/paralysis, potential causes of reflex tearing, or previous lacrimal surgery were excluded based on the clinical assessment.

NLDS was diagnosed based on patency (or partial patency) on lacrimal syringing and by the combined DCG (post-sac stenosis) and DSG (post-sac delay) findings in all eyes. Specifically, on DCG, NLDS was defined as having a duct diameter of less than that of the width of the lacrimal cannula tip on the X-ray image (27 gauge, 0.4 mm external diameter) but with patency. NLDO was confirmed by a complete post-sac block of contrast on DCG. Imaging studies were performed by trained radiologists and assessed by an experienced oculoplastic surgeon, as previously described [13].

### Procedure

All procedures were carried out or supervised by an experienced surgeon (D.S., G.D.). Powered endoscopic DCR without intubation was performed under general or local anaesthesia with sedation as previously described [14]. Briefly, the osteotomy was performed with a punch (Hajek Koffler, Martin, Tuttlingen, Germany) and powered rough-diamond burr (Medtronic-Xomed, Jacksonville, FL, USA). Mucosal apposition with the posterior lacrimal flap was ensured. Tubes were not inserted in these procedures. Postoperative instructions included daily nasal douching with a saline spray for two weeks.

### Success of intervention

Epiphora resolution was determined based on the final postoperative assessment and was scored as follows: (1) complete resolution of epiphora; (2) partial resolution; and (3) no resolution or worsening of epiphora. The intervention success assessment was based on the last postoperative follow-up before a secondary procedure if subsequent surgical procedures were undertaken.

A telephone questionnaire was conducted in patients with NLDS to evaluate longer-term postoperative improvement in epiphora. The patients were asked to quantify their symptoms as follows: (1) complete resolution of epiphora; (2) significant improvement; (3) slight improvement; (4) no change; and (5) worsening of epiphora. Patients were also asked if they would recommend DCR to others suffering from watery eyes. Those that underwent subsequent surgical procedures to treat epiphora were also contacted and were analysed separately.

### Statistical analysis

Data were analysed by the StatSoft Statistica software, version 10 (StatSoft, OK, USA). Means were compared by Student’s t-test. Proportions were compared by the chi-square or Fisher exact test, as appropriate. A two-sided p-value < 0.05 was considered significant.

## Results

A total of 24 consecutive symptomatic eyes/lacrimal systems of 23 patients (69.6% females) with NLDS that underwent endo-DCR as the primary procedure for epiphora were included. Fifty-eight consecutive lacrimal systems of 56 patients (69.6% females) with complete NLDO that underwent endo-DCR were used as a comparison group. The mean age in the NLDS and NLDO groups was 61.0 ± 17.07 (range 18–78) and 61.9 ± 17.4 (range 19–87), respectively (*p* = 0.84).

The postoperative follow-up was a mean of 13.0 ± 16.9 (range 1–84) months for cases with NLDS and 9.2 ± 10.0 (range 1–48) months for NLDO (*p* = 0.21). Resolution of epiphora was achieved in 10 (41.7% [95% CI 0.24-0.61]) of the NLDS cases compared to 40 (69.0% [95% CI 0.56–0.79]) in NLDO (*p* = 0.021). The number of cases with an improvement of epiphora (i.e., either improvement or resolution) was 17 (70.8% [95% CI 0.51–0.85]) in NLDS and 53 (91.4% [95% CI 0.81–0.96]) in NLDO (*p* = 0.034).

Anatomical patency assessment was available for six of the seven NLDS cases with no improvement in epiphora following the DCR. Of these, five had a patent ostium (i.e., functional failure), and one had internal ostium obstruction due to scarring seven months postoperative (anatomical failure).

Three patients (12.5%) with NLDS (and no postoperative improvement) underwent subsequent lacrimal procedures at a median of 14 (range 11–21) months. Of these, one (the case with internal ostium obstruction) underwent a DCR revision and subsequently improved. One patient (with functional failure) had a secondary tube insertion 16 months post-DCR, and 12 months later had insertion of a Jones tube (despite anatomical patency), reporting no improvement at the final follow-up. The third patient, similarly with functional failure, had punctal snip procedure performed 11 months post-DCR, but went on to have insertion of a Jones tube 8 months following this and improved on his final follow up. One patient (1.7%) with NLDO underwent DCR revision surgery sixteen months after the primary DCR, which resolved the epiphora.

All cases with NLDS were contacted for a phone questionnaire. Fourteen patients (15 eyes) responded, representing a 71.4% response rate. The mean time from the DCR to the phone audit was 84.0 ± 30.2 (range 7–115) months. Of these, 46.7% reported resolution (*n* = 3) or significant improvement (*n* = 4), five reported slight improvement (33.3%), and one patient reported no change. Nine (64.3%) respondents said they would recommend the DCR procedure to others suffering from watery eyes.

Finally, the three NLDS patients who underwent a subsequent lacrimal procedure (following DCR) were also contacted. Both patients that had Jones tube insertion reported no improvement (69 and 103 months postoperatively, respectively), and the patient that underwent DCR revision reported significant improvement (29 months postoperatively).

## Discussion

We found that endo-DCR in NLDS successfully resolved epiphora in 42% of cases, and overall improvement was achieved in 71%. Furthermore, 13% of all NLDS patients requested further surgical intervention due to insufficient improvement and underwent a revision or eventual insertion of a Jones tube. At the final clinical follow-up, the post-DCR resolution rate in complete NLDO (69%) was more than 1.5-fold that of NLDS, and the overall improvement rate in NLDO was higher (91%). Therefore, the results of this audit suggest that, in our hands, endo-DCR is less effective in anatomical stenosis when compared with complete anatomical obstruction.

In our long-term phone questionnaire (median close to 8 years postoperative), 64% of the NLDS patients that responded attested that they would recommend the DCR procedure to others. Taken together, based on this audit, it seems that close to 70% of patients with NLDS may benefit from endoscopic-DCR, confirming its value in this scenario.

One previous study reported the outcomes of endoscopic DCR in “functional NLDO” and in complete anatomical NLDO. Brewis et al. [12] defined ‘functional NLDO’ as patency to syringing but with evidence of delay on DSG. They did not perform DCG. As the authors themselves acknowledge, this diagnostic methodology combines NLD stenosis and non-anatomical functional delay in the “functional” cohort. The authors report the resolution of epiphora in 65% of endo-DCRs in functional NLDO and 89% in anatomical (complete) NLDO. Although the resolution rate in the “functional” group was higher than herein reported in NLDS, similar to our finding, their figures represent a significantly higher success rate for the intervention in complete anatomical NLDO than in the combined partial NLDO cohort.

Cho et al. [15] used a similar definition of ‘functional NLDO’, and reported resolution of epiphora in 81% of endo-DCRs in their cohort of combined partial NLDO. In the study of Delaney and Khooshabeh [10], ‘partial NLDO’ was also diagnosed based on clinical assessment (patency on syringing, a negative Jones I and positive Jones II test) and postsac delay on DSG. They found 80% significant improvement or resolution three years after external DCR. In both studies, no comparison was made with complete anatomical NLDO.

All three of the above studies routinely used silicone intubation in the DCRs performed in their cases of partial NLDO [10, 12, 15]. In contrast, based on our experience in primary NLDO [14], we do not routinely use tubes during DCR in cases of NLDS without canalicular abnormality. Perhaps this may contribute to the current study’s relatively lower success rates in NLDS. However, a direct comparison with these studies is also precluded by the fact that they did not assess a homogenous cohort of NLDS.

Only one study investigated the effect of DCR (external, with tubes) on NLDS and non-anatomical functional block (i.e., confirmed by DCG and DSG) separately. Peter and Pearson [16] reported that 72% of eyes with anatomical abnormalities on DCG (i.e., NLDS) had significant improvement or resolution of epiphora compared with 54% of those with normal anatomy (i.e., functional). The difference was not statistically significant. Their post-DCR improvement rate among cases of NLDS compares favourably with our finding.

Finally, our patients with NLDS were of a comparable age to those with NLDO. This contrasts with the series of Sahlin & Rose [9], in whom the partial NLD obstruction group was younger. The authors did not distinguish between NLDS and functional NLD delay in their study, as the diagnosis was based only on a patent lacrimal syringing [9]. Thus, it would be difficult to compare the results of the two studies. Nonetheless, their observation of age differences, together with the fact that drainage capacity for many of their partial NLDO patients with persistent epiphora after (external) DCR was greater than the normal capacity, may suggest that this group is disadvantaged by other factors. One possible factor is that they are hypersecretors relative to their age [17]. It is also possible that in partial NLDO, the evolution of stenosis may be related to a chronic (idiopathic) inflammatory process [10, 12]. These pathogenic mechanisms (influencing either the tear production or drainage aspects) would fit with an evolution of the dysfunctional lacrimal system over the years [17].

In conclusion, this study is the first to compare the success of endoscopic DCR in NLDS and complete NLDO, utilizing clinical assessment and comprehensive lacrimal imaging (DCG and DSG) to ensure homogenous cohorts (and notably excluding non-anatomical functional block). The results of this single-centre audit suggest that improvement in epiphora may be expected in approximately 70% of endo-DCRs in NLDS, while in complete NLDO, improvement following endo-DCR was higher (91%). These findings may assist the clinician in counselling patients regarding outcomes of endo-DCR in the context of radiologically confirmed NLDS.

### Summary

#### What was known before

- Patients with patent but dysfunctional NLD drainage are commonly encountered in the lacrimal clinic.
- Previous studies that did not methodically distinguish between nasolacrimal duct stenosis (NLDS) and non-anatomical functional block report between 50–94% success rates of DCR in this combined group of patients.

#### What this study adds

- This study is the first to compare the success of endoscopic DCR in NLDS and complete NLD obstruction, utilizing clinical assessment and comprehensive lacrimal imaging (DCG and DSG) to ensure homogenous cohorts (and notably excluding non-anatomical functional block).
- The results suggest that improvement in epiphora may be expected in approximately 70% of endo-DCRs in NLDS, while in complete NLDO, improvement following endo-DCR was significantly higher (91%).

## Data Availability

The datasets generated during and/or analysed during the current study are available from the corresponding author on reasonable request.
